# Morbidity and Mortality of Eastern Barn Owls (*Tyto javanica*) Admitted to a Southeast Queensland Wildlife Hospital

**DOI:** 10.3390/vetsci12030284

**Published:** 2025-03-18

**Authors:** Robert Doneley, Ashleigh Hicks, Andrew Hill

**Affiliations:** 1 School of Veterinary Science, The University of Queensland, Gatton, QLD 4343, Australia; ashleigh.hicks87@gmail.com; 2 Currumbin Wildlife Hospital, Currumbin, Gold Coast, QLD 4223, Australia; ahill@cws.org.au

**Keywords:** eastern barn owl, *Tyto javanica*, *Tyto alba*, wildlife, veterinary, anthropogenic

## Abstract

The eastern barn owl (*Tyto javanica*), a sub-group of *Tyto alba*, the most common species of owl in the world, is classified as “of least concern” under Queensland (Australia) legislation but appears to be affected by urban development. Between August 2010 and December 2021, 412 wild eastern barn owls were presented for treatment to the Currumbin Wildlife Hospital. A retrospective study of their medical records shows a strong association between the season and the number of admissions, with a combined total of 80% of admissions occurring in winter and spring (the breeding season for this species) in southeast Queensland. There was no significant association between age and admissions. The most common diagnosis was traumatic injuries, especially in winter and spring. Of all admissions, 46.61% died or were euthanized, 36.17% were transferred to wildlife carers, and 13.83% were discharged from the hospital. We postulate that the breeding season results in an increase in the owls’ activity, in turn resulting in greater risk exposure and increased admissions to veterinary clinics. These findings confirm that and thus provide information about the vulnerability of this species to anthropogenic activities and the implications for outcomes of injured owls.

## Simple Summary

Veterinary wildlife hospitals play a key role in wildlife conservation by not only providing care for wild animals but also recording information about the factors potentially contributing toward a decline in their populations. This paper highlights this role by documenting the anthropogenic and other risk factors affecting eastern barn owls (*Tyto javanica*) in southeast Queensland, Australia, by reviewing veterinary admission records of these owls to a regional wildlife hospital. Peak admissions occurred during the owls’ breeding season (winter and spring); traumatic injuries (fractures and soft tissue) were overall the most common reason for admission; and while the reason for admission was not reflected in the diagnosis, there was a close relationship between diagnosis and outcome. The overall finding was that anthropogenic activities can have direct effects on the viability of the eastern barn owl.

## 1. Introduction

The eastern barn owl (*Tyto javanica*), found in southeastern Asia and Australasia, is a reclusive species whose preference for secluded roosting sites makes it susceptible to urban development [1,2,3,4]. It is not commonly observed but is a frequent admission at wildlife hospitals in southeast Queensland. In the face of concerns about declining populations of eastern barn owls, like those reported for populations of barn owl (*Tyto alba*) in Europe and North America [5], and with limited feasibility for detailed field surveys, wildlife hospital records may provide valuable information about the factors influencing decline [6].

This paper reviews barn owl admissions to the Currumbin Wildlife Hospital in southeast Queensland, Australia, over a 10-year period (2010–2021). The findings provide information about human–wildlife interactions, their implications for conservation, and the effectiveness of veterinary intervention.

## 2. Materials and Methods

### 2.1. Aims

The primary aim of this study was to identify trends in hospital admission rates, diagnoses, and outcomes for eastern barn owls associated with season, age, and sex. A secondary aim was to examine whether and how the diagnosis, treatment, and length of stay in the hospital affected the outcomes for admitted birds.

### 2.2. Methods

Medical records for 412 wild eastern barn owls presented to the Currumbin Wildlife Hospital (CWH) by members of the public, wildlife carers, and other veterinarians in southeast Queensland between 3 August 2010 and 31 December 2021 were analyzed.

- The extracted patient information included (where known) sex; age; date of admission; reason for admission; diagnosis; hospital stay length; and outcome (died [euthanized, spontaneous death, unexpected death], discharged [discharged to wildlife carers, discharged into wild from the hospital], or in hospital [receiving treatment or in rehabilitation at the time data were collected]).
- The reason for admission was categorized to allow comparisons between admission variables: ground find; animal attack; motor vehicle; environmental; referral by a wildlife carer or other veterinarian; and unknown.
- The breeding season was defined as winter (July to September) and spring (October to December). The non-breeding season was defined as summer (January to March) and autumn (April to June).
- Age was classified as either adult or subadult.

Many of the responses recorded in two columns of the raw tabulated data were sorted and re-classified into set categories for the purposes of completing a valid data analysis (Supplementary Materials Table S1).

The data were examined using Microsoft 365 Excel v.2209 for sorting and counting frequencies and GraphPad Prism v9.4.1 for analysis. The categorical data were paired (as shown in Supplementary Materials Table S2) and analyzed, using the Chi-square test and Fisher exact test, to determine the effects of variables. ‘Stay length’ was analyzed against season, age, outcome (died, discharged, or in hospital), diagnosis (trauma [fracture], trauma [soft tissue], or other), and reason for admission (ground find, animal attack, human activity, or other) using an unpaired Mann–Whitney U-Test.

#### Study Limitations

The limitations to this study included the following:

- The data may have been incomplete due to a lack of standardized recording by different hospital staff.
- The small sample sizes of some categories required further collapse of categories to obtain meaningful interpretations.
- The data had low specificity for the cause of injuries and separated diagnoses in a way that may have attributed injuries from the same source into several categories. For example, motor vehicle injuries can result in soft tissue trauma; some environmental interactions such as window impact can result in bone fractures.
- The difficulties of identifying age and sex in this species, especially in a hospital setting, meant that the ages and sexes of many of the owls were not accurately determined at the time of examination. Records of sex identification were incomplete to the extent that we excluded them from the study.
- As the data were derived from a single wildlife hospital, they may not be a true measure of population dynamics.

## 3. Results

### 3.1. Seasonality

There was a strong association between the season and the number of admissions (winter 32%, spring 48%, summer 8%, and autumn 12%), with a combined total of 80% of admissions occurring in winter and spring (the breeding season for this species).

### 3.2. Age

**Figure 1:**
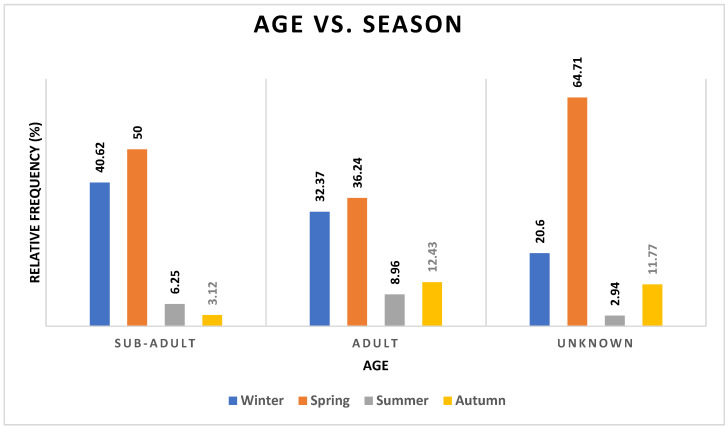
Frequencies of age categories of barn owls admitted to CWH, sorted by time of year (represented by season). See Supplementary Materials Table S3.

Adults (80%) comprised the majority of admissions overall compared to subadults (8%) and unknown (8%). Age did not appear to be related to any other category, including seasonality (*p* = 0.1655, odds ratio = 2.63) (Figure 1, Supplementary Materials Table S3).

### 3.3. Reason for Admission

The most common reason for admission was ‘ground find’ (45.87%), followed by ‘animal attack’ (26.21%) and ‘human activity’ (21.35%) (Supplementary Materials Table S4).

**Figure 2:**
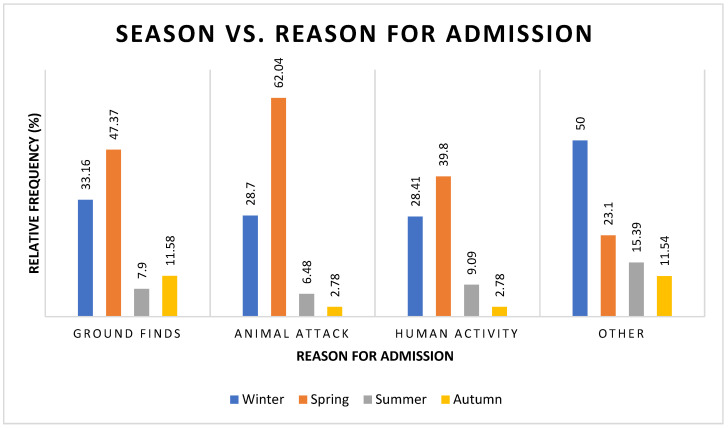
Frequency percentages of barn owls admitted to CWH sorted by season and admission history categories.

There was a statistically significant component of seasonality in the reason for admission (χ^2^ = 31.16, df = 9, *p* = 0.0003) (Figure 2, Supplementary Materials Table S4). Ground finds were presented at similar rates to overall admissions, being most common in winter and spring but less common in summer and autumn. Animal attacks were most common in spring, followed by winter, but uncommon in summer and winter. Compared to overall admissions, animal attacks occurred more frequently in spring and less frequently in autumn. Admissions due to human activities occurred more frequently compared to overall admissions in autumn, although the peak admissions due to human activities occurred in spring (n = 35).

### 3.4. Diagnosis

**Figure 3:**
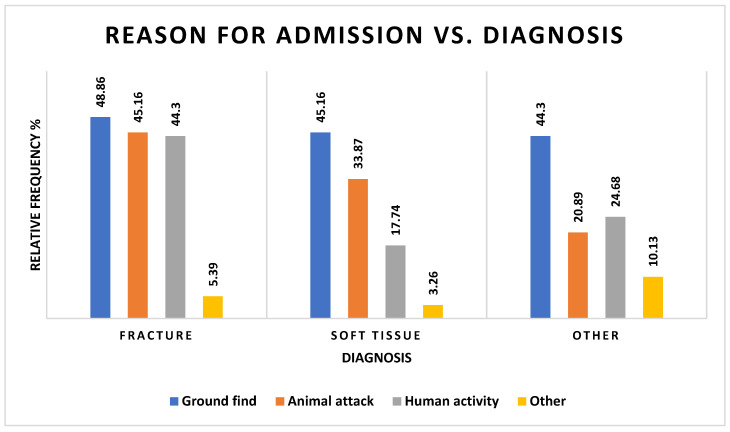
Frequencies of barn owls admitted to CWH sorted by admission history and diagnosis categories (see Supplementary Materials Table S5).

Despite approaching significance (χ^2^ = 11.85, df = 6, *p* = 0.0654), there was no association between the reason for admission and the diagnosis. Each reason for admission had similar rates of diagnosis with ‘other’ (Figure 3 and Supplementary Materials Table S5).

**Figure 4:**
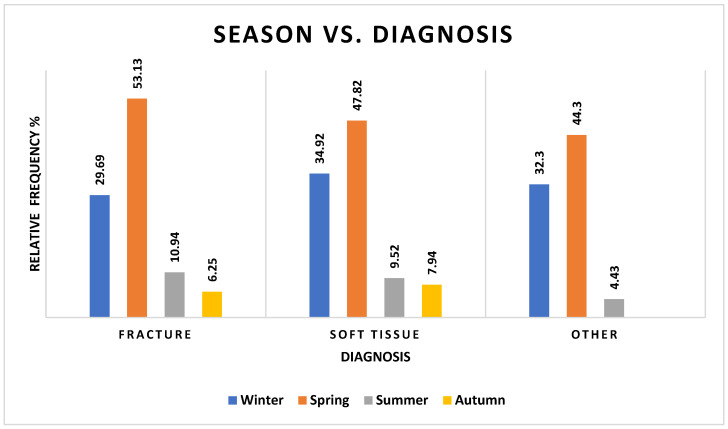
Frequencies of barn owls admitted to CWH sorted by season and diagnosis categories (see Supplementary Materials Table S6).

There was a statistically significant component of seasonality in the final diagnosis (χ^2^ = 17.94, df = 6, *p* = 0.0064). Admissions for traumatic injuries (fractures and soft tissue) in winter and spring were quite close to what was expected (i.e., more common), with fewer fractures and soft tissue injuries in autumn than expected, and more diagnoses categorized as “other” in autumn than expected (Figure 4 and Supplementary Materials Table S6).

### 3.5. Outcome

The overall frequencies of outcomes after presentation showed that 46.61% of all admissions died or were euthanized, 36.17% were discharged to wildlife carers (final outcome unknown), and 13.83% were discharged into the environment by CWH staff.

**Figure 5:**
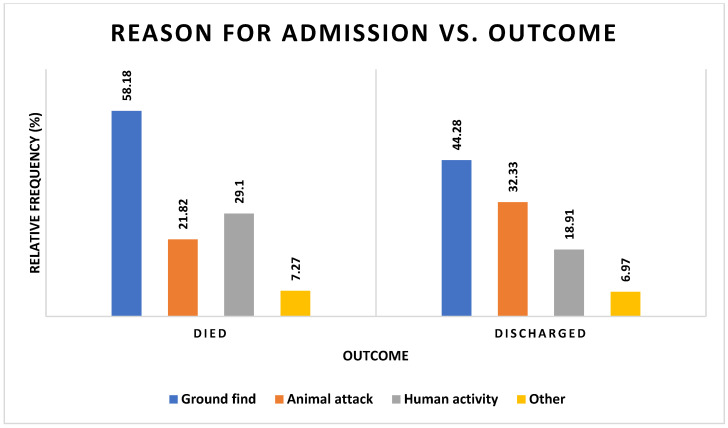
Frequencies of barn owls admitted to CWH sorted by admission history and outcome categories. Totals and percentage frequencies included. Statistical analysis included in Supplementary Materials Table S2.

There was a moderately strong relationship identified between the reason for admission and the outcome (χ^2^ = 9.427, df = 3, *p* = 0.0241) (Figure 5, Supplementary Materials Table S7).

**Figure 6:**
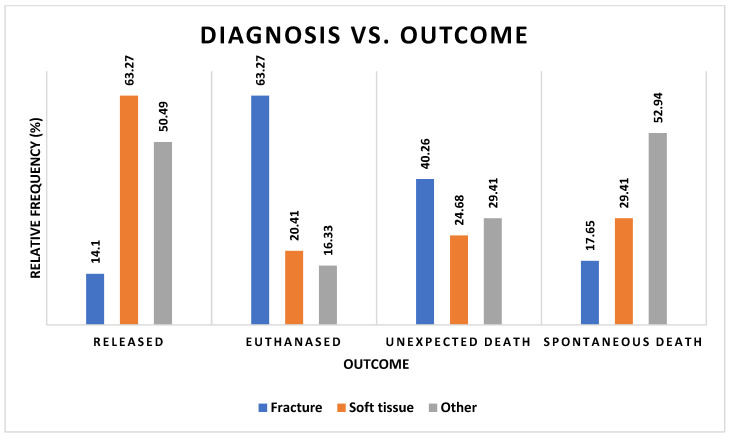
Frequencies of barn owls admitted to CWH sorted by diagnosis and outcome categories.

Diagnosis was also a very strong predictor of outcome, regardless of whether it was compared only by result of death or discharge or by manner of death or discharge (Figure 6, Supplementary Materials Table S8) (χ^2^ = 60.39, df = 2, *p* < 0.0001). Euthanasia was a more frequent outcome for fractures but was less frequent in soft tissue injuries, which had the highest relative frequency for release. ‘Other’ causes of admission also more frequently resulted in release.

**Figure 7:**
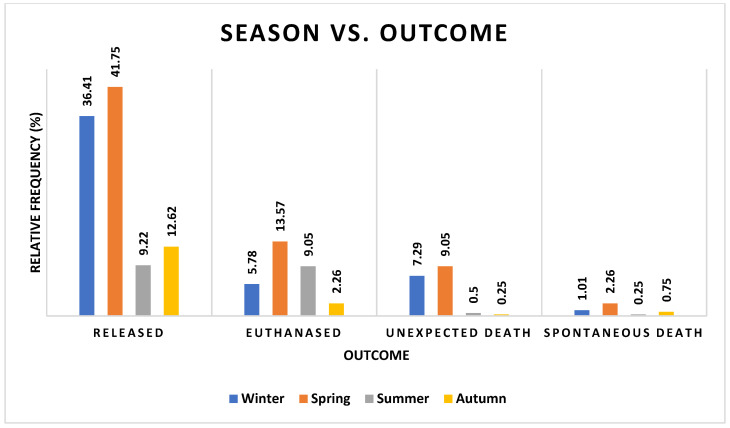
Frequencies of barn owls admitted to CWH sorted by season and outcome categories.

There was a close, but not significant, association between season and outcome (Figure 7, Supplementary Materials Table S9).

The difference between death and discharge within each diagnosis was examined and demonstrated significant differences, which are reflected in Supplementary Materials Table S8. Not surprisingly, owls that survived triage and initial stabilization spent significantly more time in care compared to those who were euthanized or died shortly after admission.

After admission and diagnosis, the stay length varied significantly according to both outcome (*p* < 0.0001, t = 11.61, r^2^ = 0.2598) and diagnosis. Birds that were eventually discharged had a median length of stay of 12 days. Regardless of the diagnosis, animals that died had shorter hospital stay lengths than did those that were discharged.

**Figure 8:**
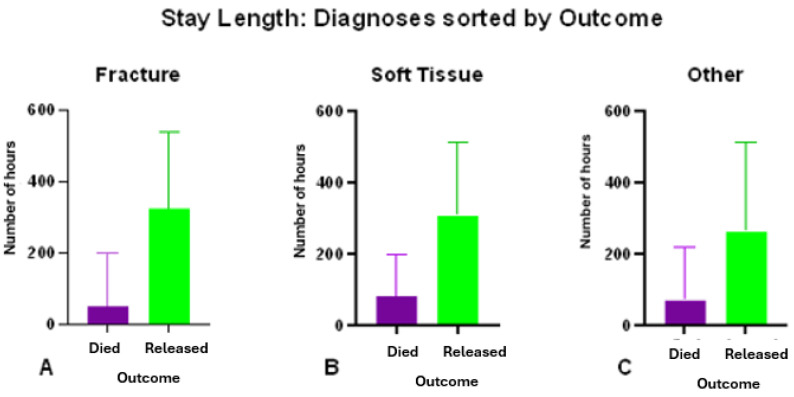
Mean stay lengths of barn owls admitted to CWH sorted into diagnoses of (**A**) fracture, (**B**) soft tissue, and (**C**) other injuries. Within diagnoses, lengths of stay are sorted by outcomes.

The median stay lengths for the different diagnoses were compared and revealed that fractures had shorter stay lengths when compared to both soft tissue injuries (*p* < 0.0001, U = 3457, n = 232) and other injuries (*p* < 0.0001, U = 5453, n = 273) (Figure 8; Supplementary Materials Table S2); however, there was no significant difference (*p* = 0.2097, U = 7919, n = 267) between stay lengths for soft tissue and other injuries.

Statistically, outcome did not have a significant association with season (χ^2^ = 20.67, df = 12, *p* = 0.0554); however, this was only marginally removed, with approximately 50% of deaths occurring in spring as opposed to the rest of the year.

## 4. Discussion

Barn owls, while globally common, can be subject to localized depletion, but how much of this population decline is a naturally occurring event, or a result of anthropogenic activities, is unknown. This study suggests that anthropogenic activities may cause an unnatural, localized depletion of barn owls.

This study demonstrated that there is compelling evidence for a seasonal trend in the number of admissions of barn owls to CWH, with most cases presented over winter and spring. We postulate that the seasonality displayed in the number and type of admissions reflects the reproductive biology of barn owls. While they can be opportunistic breeders, in subtropical regions (such as Australia), low rainfall is a significant determinant of foraging and nesting, with most breeding occurring in the dry season [1,3,7,8]. In southeast Queensland, the dry season is winter and spring (May to November), so it would be reasonable to expect that adult barn owls in southeast Queensland are likely to be more active at this time as they court, nest, mate, and raise offspring (requiring extended hunting time) and that juvenile owls lack the level of flying skills needed to avoid environmental obstacles such as cars and fences. This, in turn, would potentially result in greater risk exposure, leading to increased admissions to veterinary clinics. This result corresponds to previous findings [9,10,11,12]. As CWH sees a combined total of 80% of barn owl admissions occurring in winter and spring, it would appear this hypothesis is correct.

Surprisingly, there was little association between season and age as a predictor for admission history, diagnosis, and outcome. Since adults tend to range further into urbanized regions [13], placing them more at risk of catastrophic injury, it would be expected that they would be more likely to be presented for care at wildlife hospitals [14]. While adult birds were admitted at a higher frequency than subadults, this could be a recording bias (confusing adults and subadults) or an admission bias, as fledglings and juvenile birds do not travel as far from the nest [15,16]. As nesting sites are typically away from overt human activity, it is unlikely that dead or injured juveniles would be presented for veterinary care. It is possible that by adding data from other facilities where barn owls are treated, we may be able to increase the power of the analyses in terms of morbidity and mortality. Given that barn owls occupy a niche that will potentially be increasingly developed over time, access to these data will be invaluable for ecological analysis [17].

The hunting behavior of barn owls requires a low-lying, flat, open habitat such as grasslands, farmland, and peri-urban habitats [1,3,9,17]. With increasing urbanization in southeast Queensland, barn owls are being increasingly concentrated into peri-urban areas such as farms and re-zoned residential areas, where such habitats become less common and smaller [4,17]. Although (at first) they appear to have adapted well to urbanization [18], this habitat preference places them at risk from many human activities such as vehicle use, rodent control, predation by domestic pets, and land clearing (which often results in the loss of roosting trees) [18]. Other threats include competition for resources from other animals (such as parrots and marsupials) displaced by anthropogenic activity [19] and intermittent localized declines in food availability, e.g., the collapse of a rodent population following a mouse plague [10].

We hypothesize that these habitat changes should increase the number of traumatic injuries as barn owls encounter fences, powerlines, household pets, and motor vehicles, especially as they are less reliant on vision while flying. Our study supports this hypothesis, with traumatic musculoskeletal injuries being more common in the active breeding season.

The diagnosis and outcome cannot be predicted from the reason for admission, but the diagnosis can be expected to predict the outcome. Although the association between season and outcome was close, it was not significant. Despite this, approximately 50% of deaths occurred in spring, as opposed to the rest of the year seeing higher frequencies of discharge than death.

CWH triages all patients on admission, euthanizing those with a hopeless prognosis for rehabilitation and discharge. Those animals with a fair to good prognosis are admitted for ongoing care, including surgery. The outcomes after admission are death (euthanasia or because of injuries/disease) or discharge (transfer to wildlife carers [for long-term rehabilitation and discharge] or discharge by hospital staff into the wild).

Barn owls that survived triage and initial stabilization spent significantly more time in care compared to those who were euthanized or died shortly after admission. Although the median stay length was only 4 days, for the 119 barn owls that were admitted for hospital care, the median stay in care was 269 days. Road traumas do not always present as fractures, and soft tissue injuries such as ocular lesions may hinder the bird’s ability to hunt [20]. The median stay lengths of birds with soft tissue injuries were similar to those of birds with fractures.

Within the scope of this study, we cannot make inferences beyond identifying that diagnosis is likely to be a predictor of death in barn owls admitted to veterinary care. When one considers that previous studies, like our own, have found that the majority of injuries are fractures and that those fractures are mostly from road accidents [15], it makes sense that catastrophic injury has a positive relationship with predicting death. This is an important finding for the purposes of our study, as it further cements the need to try to mitigate injuries from interactions between barn owls and human activities; as these owls are dwellers of peri-urban and grassland habitats, in the context of ever-expanding urbanization, planning is the best prevention.

An unexpected finding in our study was that age was not a predictor of admission history, diagnosis, or outcome since adults tend to range further into urbanized regions, placing them more at risk of catastrophic injury [13]. This could represent a recording bias; it can be difficult to differentiate age groups in this species, and, as this was a retrospective study, age was often not recorded in medical records. The frequencies of diagnosis showed an even spread across diagnoses, with around 30% of each for adults and 3% of each for subadults, and we know that there were approximately ten times more adults admitted than subadults, so the lack of association appears probable. When compared by age, the frequencies of outcome followed a similar pattern. Of course, as previously mentioned when discussing admission, death rates by age are difficult to quantify since many birds will be neither found nor reported.

Whether comparing diagnosis to condensed outcomes of either death or recovery, we see a very significant relationship between the two variables, and we again see evidence for a relationship when utilizing all of the categorical labels of outcome (spontaneous death, euthanasia, unexpected death, discharged to care or the wild). Delving deeper into this, we found that while diagnosis did not have a relationship with whether barn owls were discharged straight from the clinic back to the environment or whether they were discharged to care (either with a carer or in the raptor aviary at CWH), there was a significant relationship with whether death occurred while in care, via humane euthanasia, or due to an unexpected cause (a reporting issue).

### Future Directions

The predator density in any geographical region tends to be low; when the animal in question is also nocturnal and cryptic, gathering information about population wellbeing is especially difficult [19]. Retrospective studies may be the only practical means for obtaining such information relating to population morbidity and mortality. This study’s results correlate well with previously published necropsy investigations into deaths in barn owls from Australia and the United Kingdom [10,11,12], which found that many deaths were associated with motor vehicle impacts, and that there was a component of seasonality attached, likely aligned with the breeding season.

Retrospective necropsy studies, which are both objective and quantitative, may offer a better measure of causes of mortality than retrospective clinical studies due to incomplete or incorrect data that can lead to errors in analysis and interpretation. Defining categories and outlining classification methods for recording staff would contribute to uniformity of recording and better data for analysis. However, necropsy studies, by definition, do not include live animals and so may be incomplete in the depth and breadth of the data collected.

We speculate that the way our data were categorized may not allow us to make proper inferences about how often human activities were involved in the injuries sustained. If we could more accurately link road-related injuries with a certain time of year or pressure, then perhaps recommendations could be made to councils about how to help negate this occurrence. Certainly, we were encouraged to see that there was a component of seasonality to both admission and diagnosis, as expected, although we cannot say whether this followed foraging pressure or some other variable within the scope of this study; in the future, this can be ascertained.

Unfortunately, sex as a determinant of outcomes after admission to CWH was expected to give a large amount of insight into the life of eastern barn owls of southeast Queensland; we wanted to determine the shape of the relationship for both males and females, how they compared to each other, and how they compared to the overall distribution of wild barn owls in the region. Without a significant portion of the data, we were unable to explore this; if these data can be properly recorded in future, this is likely to be a valuable investigation.

A more targeted study of ecological changes over time would also be interesting and potentially useful for town planning. As urban areas begin to encroach on grassland and farmland habitats, animals are known to be increasingly restricted in terms of their viable hunting land. Sampling from more veterinary facilities to gain a perspective on whether new roads and the incursion of suburban sprawl increase motor vehicle accidents, for instance, may help with council planning of ecological corridors designed to provide hunting areas away from roads so that we can attempt to mitigate traumas.

Ecological surveys of feeding behavior in southeast Queensland would also potentially yield important insights into the conservation of sufficient DNA variability from breeding pairs. While barn owls are currently legislatively rated as a ’least concern‘ species, they are important apex predators of small mammals and lizards, making a sustainable population an important environmental goal.

## 5. Conclusions

The information collected by CWH over the last decade shows that there is a seasonal association for barn owl admissions and an association between the diagnosis and outcome. This seasonal association reflects the barn owl breeding season, suggesting that increased activity by both adults and juveniles may result in more injuries and disease.

There may be a seasonal association with the age and sex of patient admissions, but the practical difficulties in identifying this in individual birds make it difficult to be confident in stating this association.

Treatment of injured barn owls is possible even when they have sustained fractures, and they can and do return to the wild with a median time in care of under one year.

The information collected and collated by CWH is an important source of data that might aid town and road planning, so that even as urbanization encroaches into the owls’ territory, councils might have a chance to create mitigation strategies to hopefully reduce the rate of injuries in the population. To aid future analyses, a formal standard operating procedure for examining the owls should be considered to collect all relevant information, making the data more robust and ensuring that they can be utilized to full effect to help reduce the morbidity and mortality of this species and maintain its position as an important apex predator.

## Data Availability

All data are contained within this manuscript and Supplementary Materials.

## Supplementary Materials

The following supporting information can be downloaded at: https://www.mdpi.com/article/10.3390/vetsci12030284/s1, Table S1. Reclassification of data into set categories for data analysis. Table S2. Compilation of reportable comparative statistics analyzing frequency tables of barn owls admitted to CWH, to determine strength of associations, if any. Significance (≤0.05) denoted by stars * low, ** moderate, *** high, **** very high. a = died or released; b = died (spontaneous, euthanasia, unexplained) or released (to the wild, to a carer). Table S3. Frequency of age of barn owls admitted to CWH sorted by time of year (represented by season). Statistical analysis included in Table S2. AF = absolute frequency; RF^1^ = relative frequency in each age group by season, RF^2^ = relative frequency in total population by season. Table S4. Frequency of barn owls admitted to CWH sorted by season and Reason for admission categories. Totals and percentage frequency included. Statistical analysis included in Table S2. AF = absolute frequency; RF = relative frequency. Table S5. Frequency of barn owls admitted to CWH sorted by Reason for admission and Diagnosis categories. Totals and percentage frequency included. Statistical analysis included in Table S2. AF = absolute frequency; RF = relative frequency. Table S6. Frequency of barn owls admitted to CWH sorted by season and Diagnosis categories. Totals and percentage frequency included. Statistical analysis included in Table S2. AF = absolute frequency; RF = relative frequency. Table S7. Frequency of barn owls admitted to CWH sorted by Reason for admission and outcome categories. Totals and percentage frequency included. Statistical analysis included on Table S2. Table S8. Frequency of barn owls admitted to CWH, sorted by diagnosis and outcome categories. Totals and percentage frequency included. Statistical analysis included in Table S2. Table S9. Frequency of barn owls admitted to CWH sorted by season and outcome categories. Totals and percentage frequency included. Statistical analysis included on Table S2.
