# Supplementary material for: Morbidity and Mortality of Eastern Barn Owls (Tyto javanica) Admitted to a Southeast Queensland Wildlife Hospital
Source: Vet Sci. 2025 Mar 18;12(3):284. doi: 10.3390/vetsci12030284 (PMC11946146; doi:10.3390/vetsci12030284)
Supplement: Supplementary file 1 [file vetsci-12-00284-s001.zip › vetsci-3353327-supplementary.pdf]

Supplementary Materials

Morbidity and Mortality of Eastern Barn Owls (*Tyto javanica*) Admitted to a Southeast Queensland Wildlife Hospital

Table S1. Reclassification of data into set categories for data analysis.

Column label	Initial Response	Converted Response	Condensed Response
Reason for admission	Apparently stunned, apparently weak, dead-on-arrival, fallen from tree, found beside road, found on ground away from road, found on road, ground find, limb or limbs dragging, orphaned or (n) adults nearby, reported ill, road find, unable to fly, unable to stand up, unable to walk, water soaked, wing drooping or dragging	Ground find	NA
	Listed as attacked by any animal	Animal Attack	NA
	Hit by vehicle (presumed), vehicle hit	Motor Vehicle	Human activity
	Barbed wire entanglement, entanglement (barbed wire), entrapped, floating in water, snagged on object, suspected poisoning, window hit	Environment	
	Brought in by a carer, veterinarian referral, reported ill	Referral	Other
	Not known, other	Unknown	
Admission Date	Any date listed from the beginning of December to end of February in any year	Summer	Off Season
	Any date listed from the beginning of March to end of May in any year	Autumn	
	Any date listed from the beginning of June to end of August in any year	Winter	Breeding Season
	Any date listed from the beginning of September to end of November in any year	Spring	
Age	Hatchling, fledgling, post fledgling, juvenile, subadult	Subadult	NA

Table S2. Compilation of reportable comparative statistics analyzing frequency tables of barn owls admitted to CWH to determine strength of associations, if any. Significance (≤ 0.05) denoted by stars: *low, **moderate, ***high, ****very high.

Test Statistics Summary				
Test Used	Comparison		Relevant reporting	Significance
Chi-square	Season vs. Reason for admission		$\chi2 = 31.16$, df = 9 , $p = 0.0003$	***
	Season vs. Diagnosis		$\chi2 = 17.94$, df = 6, $p = 0.0064$	**
	Season vs. Outcome		$\chi2 = 20.67$, df = 12, p (≤ 0.06	(n)
	Age vs. Reason for admission		$\chi2 = 4.855$, df = 3, $p = 0.1828$	(n)
	Age vs. Diagnosis		$\chi2 = 0.2979$, df = 2, $p = 0.8616$	(n)
	Age vs. Outcome		$\chi2 = 0.2813$, df = 1, $p = 0.5958$	(n)
	Reason for admission vs. Diagnosis		$\chi2 = 11.85$, df = 6, $p = 0.0654$	(n)
	Reason for admission vs. Outcome		$\chi2 = 9.427$, df = 3, $p = 0.0241$	*
	Diagnosis vs. Outcome (simple) [a]		$\chi2 = 60.39$, df = 2, $p < 0.0001$	****
	Diagnosis vs. Outcome [b]		$\chi2 = 82.15$, df = 2, $p < 0.0001$	****
	Diagnosis vs. Release		$\chi2 = 1.304$, df = 2, $p = 0.5211$	(n)
	Diagnosis vs. Death		$\chi2 = 19.35$, df = 4, $p = 0.0007$	***
Fisher's exact	Age vs. Discharge		$p = 0.7107$, O.ratio = 0.8193	(n)
	Age vs. Breeding Season		$p = 0.1655$, O.ratio = 2.63	(n)
Mann–Whitney U	Stay length by outcome (died vs. released)		$p < 0.0001$, U = 4492, n = 386	****
	Stay length: Diagnosis by outcome (died vs. released)	fracture by outcome	$p < 0.0001$, U = 146, n = 119	****
		soft tissue by outcome	$p < 0.0001$, U = 418, n = 113	****
		other by outcome	$p < 0.0001$, U = 592, n = 118	****
	Stay length by Diagnosis	fracture vs. soft tissue	$p < 0.0001$, U = 3457, n = 232	****
		fracture vs. other	$p < 0.0001$, U = 5453, n = 273	****
		soft tissue vs. other	$p = 0.2097$, U = 7919, n = 267	(n)

a = died or released; b = died (spontaneous, euthanasia, unexplained) or released (to the wild, to a carer).

Table S3. Frequencies of age groups of barn owls admitted to CWH sorted by time of year (represented by season). Statistical analysis included in Table S2. AF = absolute frequency; RF[1] = relative frequency in each age group by season; RF[2] = relative frequency in total population by season.

	**Total (all ages)**		**Age Group**					
			**Subadult**		**Adult**		**Unknown age**	
	AF	RF (/412)	AF	RF (/32)	AF	RF (/346)	AF	RF (/34)
	n	%	*n*	%	*n*	%	*n*	%
**Winter**	132	32.04	13	40.63	112	32.37	7	20.59
**Spring**	198	48.06	16	50.0	160	46.24	22	64.71
**Summer**	34	8.25	2	6.25	31	8.99	1	2.94
**Autumn**	48	11.65	1	3.13	43	12.43	4	11.77
**Total**	412	100	32	100	346	100	34	100

Table S4. Frequencies of barn owls admitted to CWH sorted by season and reason for admission categories. Totals and percentage frequencies included. Statistical analysis included in Table S2. AF = absolute frequency; RF = relative frequency.

**Season vs. Reason for admission**										
Season	**Total admissions**		**Reason for admission**							
			Ground find		Animal attack		Human Activity		Other	
	AF	RF (/412)	AF	RF (/190)	AF	RF (/108)	AF	RF (/88)	AF	RF (/26)
	n	%	*n*	%	*n*	%	*n*	%	*n*	%
Winter	132	32.04	63	33.16	31	28.70	25	28.41	13	50.00
Spring	198	48.06	90	47.37	67	62.04	35	39.80	6	23.10
Summer	34	8.25	15	7.90	7	6.48	8	9.09	4	15.39
Autumn	48	11.65	22	11.58	3	2.78	20	22.73	3	11.54
Total	412	100	190	100	108	100	88	100	26	100

Table S5. Frequencies of barn owls admitted to CWH sorted by reason for admission and diagnosis categories. Totals and percentage frequencies included. Statistical analysis included in Table S2. AF = absolute frequency; RF = relative frequency.

**Reason for admission vs. Diagnosis**								
**Reason for admission**	**Total admissions**		**Diagnosis**					
			**Fracture**		**Soft tissue**		**Other**	
	AF	RF (/412)	AF	RF (/130)	AF	RF (/124)	AF	RF (/158)
	n	%	*n*	%	*n*	%	*n*	%
Ground find	189	45.87	63	48.46	56	45.16	70	44.30
Animal attack	108	26.21	33	25.39	42	33.87	33	20.89
Human Activity	88	21.36	27	20.77	22	17.74	39	24.68
Other	27	6.55	7	5.39	4	3.26	16	10.13
Total	412	100	130	100	124	100	158	100

Table S6. Frequencies of barn owls admitted to CWH sorted by season and diagnosis categories. Totals and percentage frequencies included. Statistical analysis included in Table S2. AF = absolute frequency; RF = relative frequency.

**Season vs. Diagnosis**								
**Season**	**Total admissions**		**Diagnosis**					
			Fracture		Soft tissue		Other	
	AF	RF (/412)	AF	RF (/128)	AF	RF (/126)	AF	RF (/158)
	n	%	*n*	%	*n*	%	*n*	%
Winter	133	32.28	38	29.69	44	34.92	51	32.30
Spring	198	48.06	68	53.13	60	47.62	70	44.30
Summer	33	8.01	14	10.94	12	9.52	7	4.43
Autumn	48	11.65	8	6.25	10	7.94	30	19.00
Total	412	100	128	100	126	100	158	100

Table S7. Frequencies of barn owls admitted to CWH sorted by reason for admission and outcome categories. Totals and percentage frequencies included. Statistical analysis included in Table S2.

Reason for admission vs. Outcome						
**Reason for admission**	**Total admissions**		**Outcome**			
			Died		Released	
	AF	RF (/366)	AF	RF (/165)	AF	RF (/201)
	n	%	*n*	%	*n*	%
Ground find	185	50.55	96	58.18	89	44.28
Animal attack	101	27.6	36	21.82	65	32.33
Human Activity	86	23.5	48	29.10	38	18.91
Other	26	7.1	12	7.27	14	6.97
Total	366	100	165	100	201	100

Table S8. Frequencies of barn owls admitted to CWH sorted by diagnosis and outcome categories. Totals and percentage frequencies included. Statistical analysis included in Table S2.

Diagnosis vs. Outcome													
**Diagnosis**		**Total admissions**		**Outcome**									
				Total deaths		Released		Euthanized		Unexplained Death		Spontaneous Death	
		AF	RF (/398)	AF	RF (/182)	AF	RF (/206)	AF	RF (/98)	AF	RF (/77)	AF	RF (/17)
		n	%	*n*	%	*n*	%	*n*	%	*n*	%	*n*	%
Trauma	Fracture	125	31.41	96	52.75	29	14.10	62	63.27	31	40.26	3	17.65
	Soft tissue	117	29.40	44	24.18	73	35.44	20	20.41	19	24.68	5	29.41
Other		156	39.20	42	23.08	104	50.49	16	16.33	27	35.10	9	52.94
Total		398	100	182	100	206	100	98	100	77	100	17	100

Table S9. Frequencies of barn owls admitted to CWH sorted by season and outcome categories. Totals and percentage frequencies included. Statistical analysis included in Table S2.

Season vs. Outcome												
**Season**	**Total admissions**		**Outcome**									
			Total deaths		Released		Euthanized		Unexplained Death		Spontaneous Death	
	AF	RF (/398)	AF	RF (/192)	AF	RF (/206)	AF	RF (/98)	AF	RF (/77)	AF	RF (/17)
	n	%	*n*	%	*n*	%	*n*	%	*n*	%	*n*	%
Winter	131	32.91	56	60.87	75	36.41	23	5.78	29	7.29	4	1.01
Spring	185	46.48	99	51.56	86	41.75	54	13.57	36	9.05	9	2.26
Summer	34	8.54	15	7.81	19	9.22	12	3.02	2	0.50	1	0.25
Autumn	48	12.06	22	11.46	26	12.62	9	2.26	10	2.51	3	0.75
Total	398	100	192	100	206	100	98	100	77	100	17	100
